# How Should We Use Hyaluronidase for Dissolving Hyaluronic Acid Fillers?

**DOI:** 10.1111/jocd.16783

**Published:** 2025-01-15

**Authors:** Gi‐Woong Hong, Hyewon Hu, Jovian Wan, Kathleen Chang, Youngjin Park, Massimo Vitale, Giovanni Damiani, Kyu‐Ho Yi

**Affiliations:** ^1^ Samskin Plastic Surgery Clinic Seoul Korea; ^2^ Division in Anatomy and Developmental Biology, Department of Oral Biology Human Identification Research Institute, BK21 FOUR Project, Yonsei University College of Dentistry Seoul Korea; ^3^ Sihler Inc. Seoul Korea; ^4^ Harmony Aesthetic Clinic Adelaide Australia; ^5^ Obliv Clinic Incheon Korea; ^6^ Private Practice Bologna Italy; ^7^ Department of Biomedical, Surgical and Dental Sciences University of Milan Milan Italy; ^8^ Italian Center of Precision Medicine and Chronic Inflammation Milan Italy; ^9^ Clinical Dermatology IRCCS Ospedale GaleazziSant'Ambrogio Milan Italy; ^10^ You & I Clinic (Mokdong) Seoul Korea

**Keywords:** biodegradation, filler complications, hyaluronic acid, hyaluronidase, injectable fillers

## Abstract

**Background:**

Hyaluronic acid (HA) fillers are commonly used in esthetic medicine for facial contouring and rejuvenation. However, complications such as overcorrection, vascular occlusion, and irregular filler distribution necessitate the use of hyaluronidase to dissolve the fillers. This study aimed to evaluate the efficacy of hyaluronidase in degrading different types of HA fillers and provide clinical guidelines for its use based on filler type, dosage, and application techniques.

**Methods:**

A series of in vitro and in vivo experiments were conducted to assess the dissolution of biphasic and monophasic HA fillers using varying concentrations of hyaluronidase. The in vivo component used animal models to determine the duration of hyaluronidase activity in biological tissues, whereas the in vitro study examined the dissolution rates of HA fillers in response to different hyaluronidase concentrations and application methods. A control study using saline was also performed to compare the natural hydration process of the fillers.

**Results:**

Hyaluronidase efficacy was found to vary based on the type of HA filler and the enzyme's concentration. Biphasic fillers dissolved more rapidly at lower concentrations of hyaluronidase compared to monophasic fillers, which required higher concentrations and longer exposure times for effective breakdown. The study also demonstrated that direct injection of hyaluronidase into the filler mass was more effective than surface application. Pharmacokinetic analysis revealed that hyaluronidase activity diminished within 30 min in biological tissues, highlighting the need for timely intervention in clinical scenarios.

**Conclusion:**

Hyaluronidase is effective in dissolving HA fillers, with its efficacy dependent on the type of filler, concentration, and application technique. Biphasic fillers respond more quickly to hyaluronidase, whereas monophasic fillers require higher doses and multiple treatments. Clinical recommendations include using direct injection techniques, tailoring hyaluronidase dosage based on the filler type, and considering hypersensitivity reactions. Future research should focus on the long‐term effects of hyaluronidase and refining clinical protocols for its use.

## 1 Introduction

The use of hyaluronic acid (HA) fillers for esthetic treatments has become widespread, but complications such as overcorrection, uneven distribution, and vascular occlusion can necessitate corrective interventions [1, 2, 3, 4]. The primary aim of this study is to evaluate the effectiveness of hyaluronidase in dissolving HA fillers in clinical settings and to establish guidelines for its optimal use. Specifically, this research focuses on how hyaluronidase interacts with different types of HA fillers, the concentrations required for effective dissolution, and the best techniques for its administration.

HA fillers used in treatments are crosslinked, a process that alters their degradation behavior and allows them to maintain their form in the body for extended periods. The degree of crosslinking varies by product, which influences how long fillers last and how they respond to hyaluronidase [5]. In clinical practice, the longevity of HA fillers is influenced by injection depth and location, with fillers near the periosteum generally lasting longer due to lower concentrations of natural hyaluronidase in deeper tissues.

Hyaluronidase is an enzyme commonly used to dissolve HA fillers when complications arise [6]. In this study, we aim to examine the pharmacokinetics of hyaluronidase and its efficiency in dissolving both monophasic and biphasic HA fillers. The molecular breakdown of HA by hyaluronidase occurs through the hydrolysis of β‐1,4 and β‐1,3 glycoside bonds, which reduces the filler into smaller fragments that are eventually excreted [6, 7, 8]. Understanding how hyaluronidase works in different clinical scenarios will help improve its application in managing filler‐related complications.

While some aspects of hyaluronidase's pharmacodynamics are well understood, such as its short half‐life in subcutaneous tissues (~30 min) and even shorter in the bloodstream (~2 min), more research is needed to determine how long its effects last after injection and how it can be best used in various clinical situations [6]. This study seeks to provide a clearer understanding of the enzyme's role in managing HA filler complications, offering clinicians insights on dosage, application techniques, and the factors that influence its efficacy.

## 2 Materials and Methods

This study involved both in vivo and in vitro experiments to evaluate the efficacy of hyaluronidase in dissolving HA fillers. The experiments were designed to assess the duration of hyaluronidase activity, as well as the differences in its effectiveness between biphasic and monophasic fillers. Additionally, a control study using saline was performed to compare the natural dissolution of the fillers. The in vivo component involved animal models, whereas the in vitro component focused on filler dissolution and hydration.

### 2.1 General Methods

**FIGURE 1:**
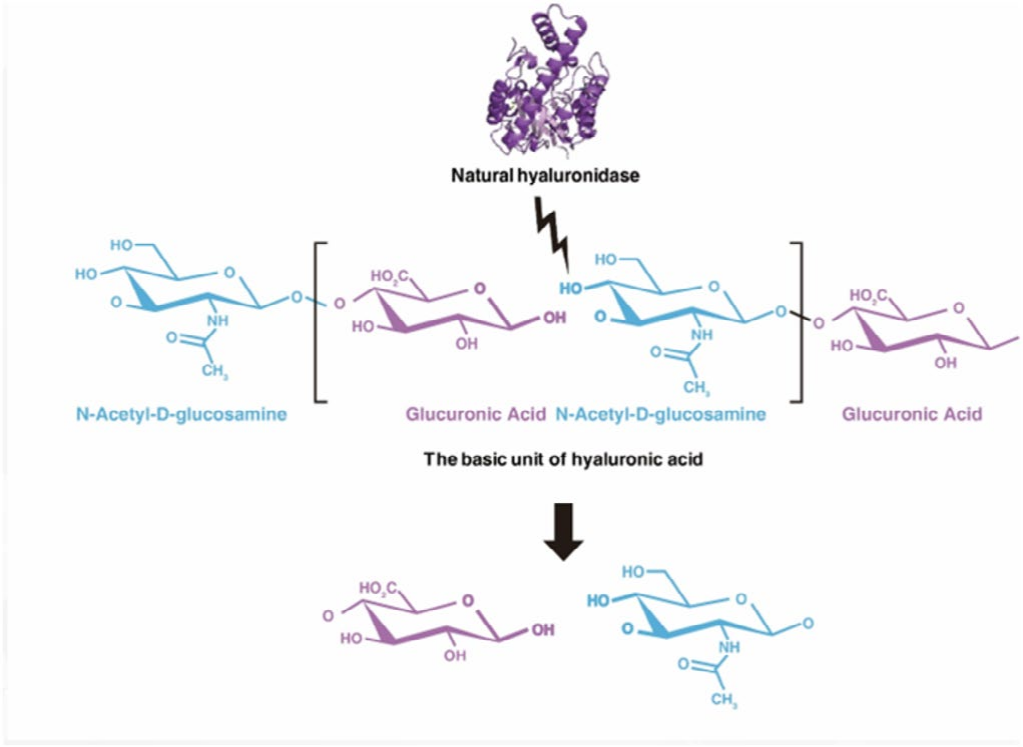
A comparison of two techniques to degrade the filler mass using 300 IU of hyaluronidase. The “drop technique” surrounds the filler with hyaluronidase solution, whereas the “injection technique” infiltrates the interior of the filler mass.

Hyaluronidase was prepared in different concentrations by diluting 1500 units in 2 or 4 mL of normal saline, creating two solutions: 75 units/0.1 mL and 37.5 units/0.1 mL, respectively. Equal masses (0.125 mL) of biphasic (Restylane Lidocaine) and monophasic fillers were extracted for the experiments. Two techniques were used for hyaluronidase application:

1. Surface application: The hyaluronidase solution was applied around the exterior of the filler mass.
2. Direct injection: Hyaluronidase was injected into the filler mass to enhance degradation (Figure 1).

The in vitro study examined the dissolution of fillers at different hyaluronidase concentrations, and the in vivo study determined the duration of hyaluronidase activity in tissue.

### 2.2 Animal Experiment to Determine Hyaluronidase Activity Duration

**FIGURE 2:**
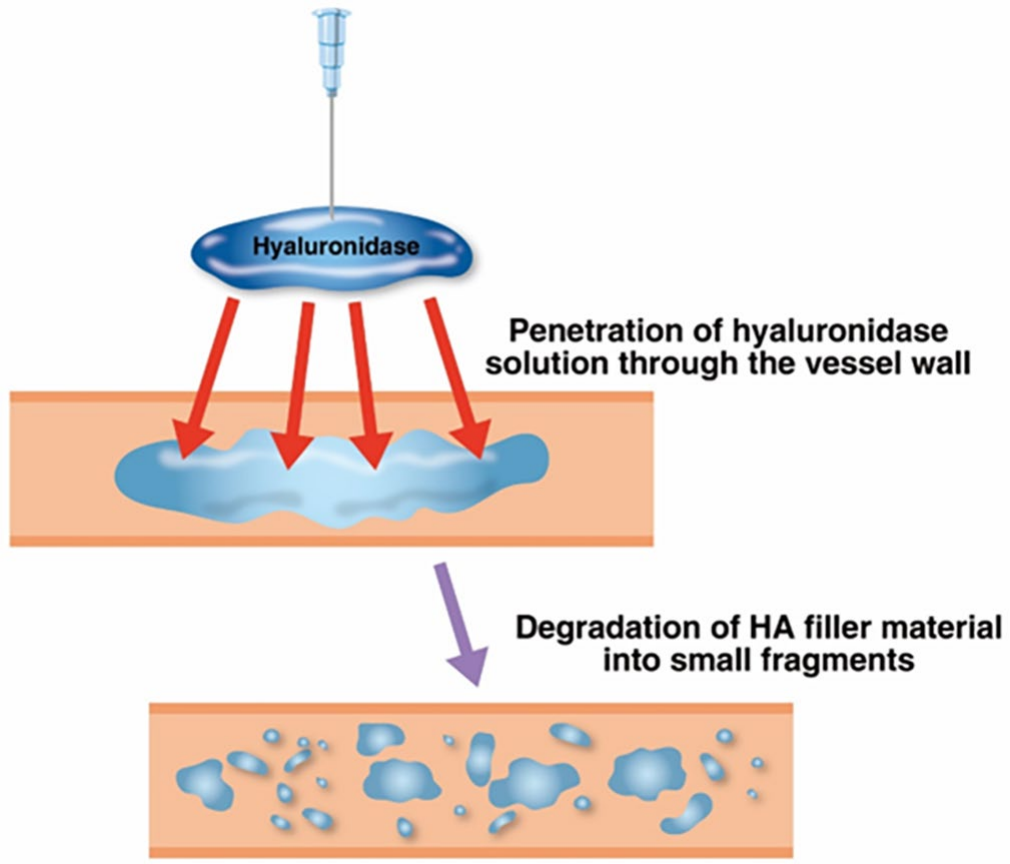
The process of hyaluronic acid (HA) filler degradation when located inside a blood vessel, as a result of hyaluronidase solution infiltration in the perivascular space.

To assess the duration of hyaluronidase activity in biological tissues, an in vivo experiment was conducted using mice. In each mouse, 0.2 mL of HA filler was injected subcutaneously, followed by the administration of 600 IU of hyaluronidase to degrade the filler. HA filler was then reinjected at intervals of 30 min, 1, 3, 6, 12, 24 h, and 2, 4, 7, and 14 days after the initial hyaluronidase injection. Tissue samples were collected at each interval to assess the effect of hyaluronidase on the reinjected filler (Figure 2).

#### 2.2.1 Results for Animal Experiment

Hyaluronidase activity diminished significantly within 30 min of injection. Reinjected filler at later intervals was largely unaffected by residual hyaluronidase, indicating that the enzyme's activity window is short‐lived. This suggests that timely hyaluronidase administration is critical for its effectiveness in degrading HA filler in clinical settings.

### 2.3 In Vitro Dissolution Experiments on Biphasic and Monophasic Fillers

**FIGURE 3:**
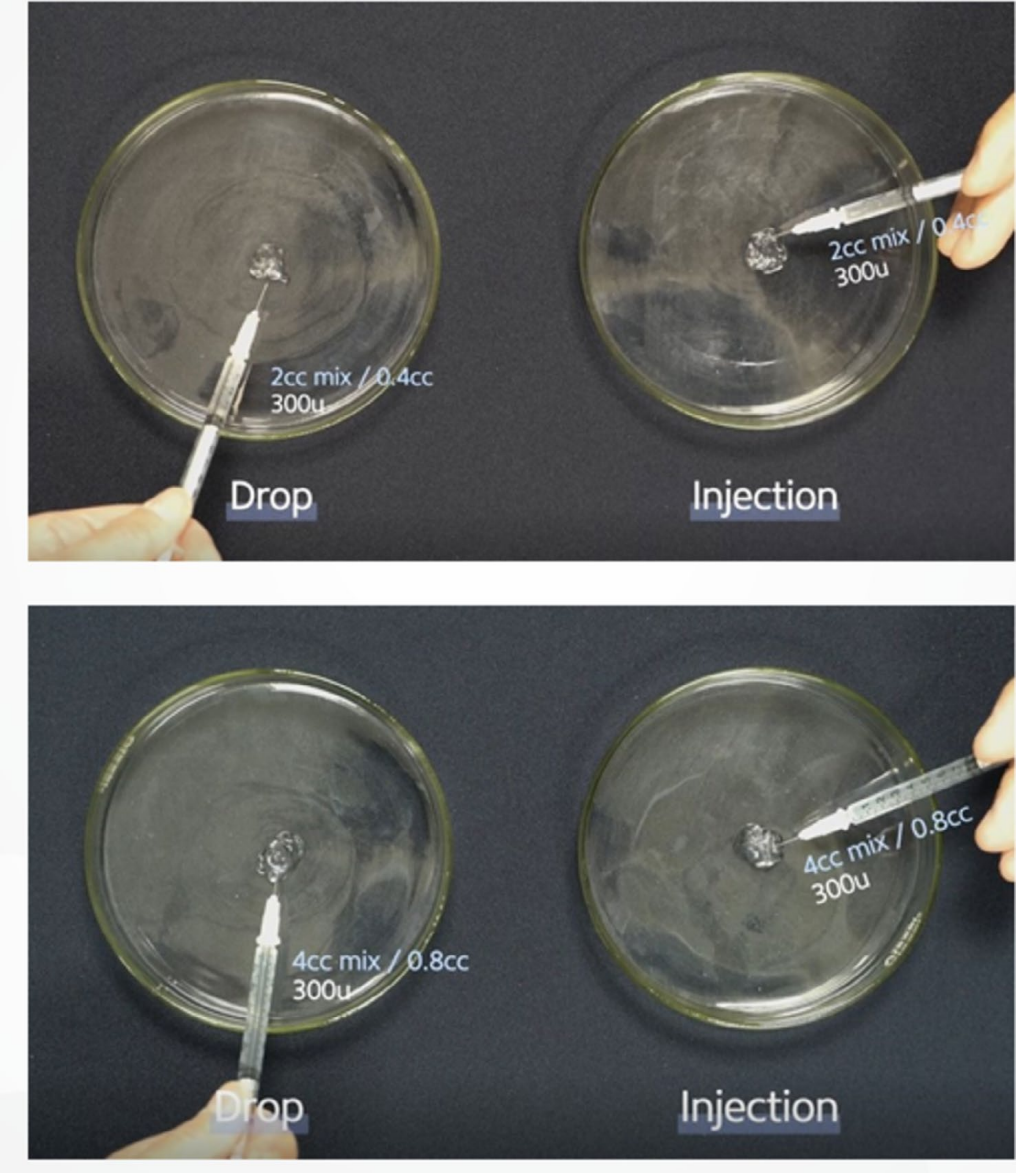
Hydration and dissolution of biphasic HA fillers treated with varying concentrations of hyaluronidase.

**FIGURE 4:**
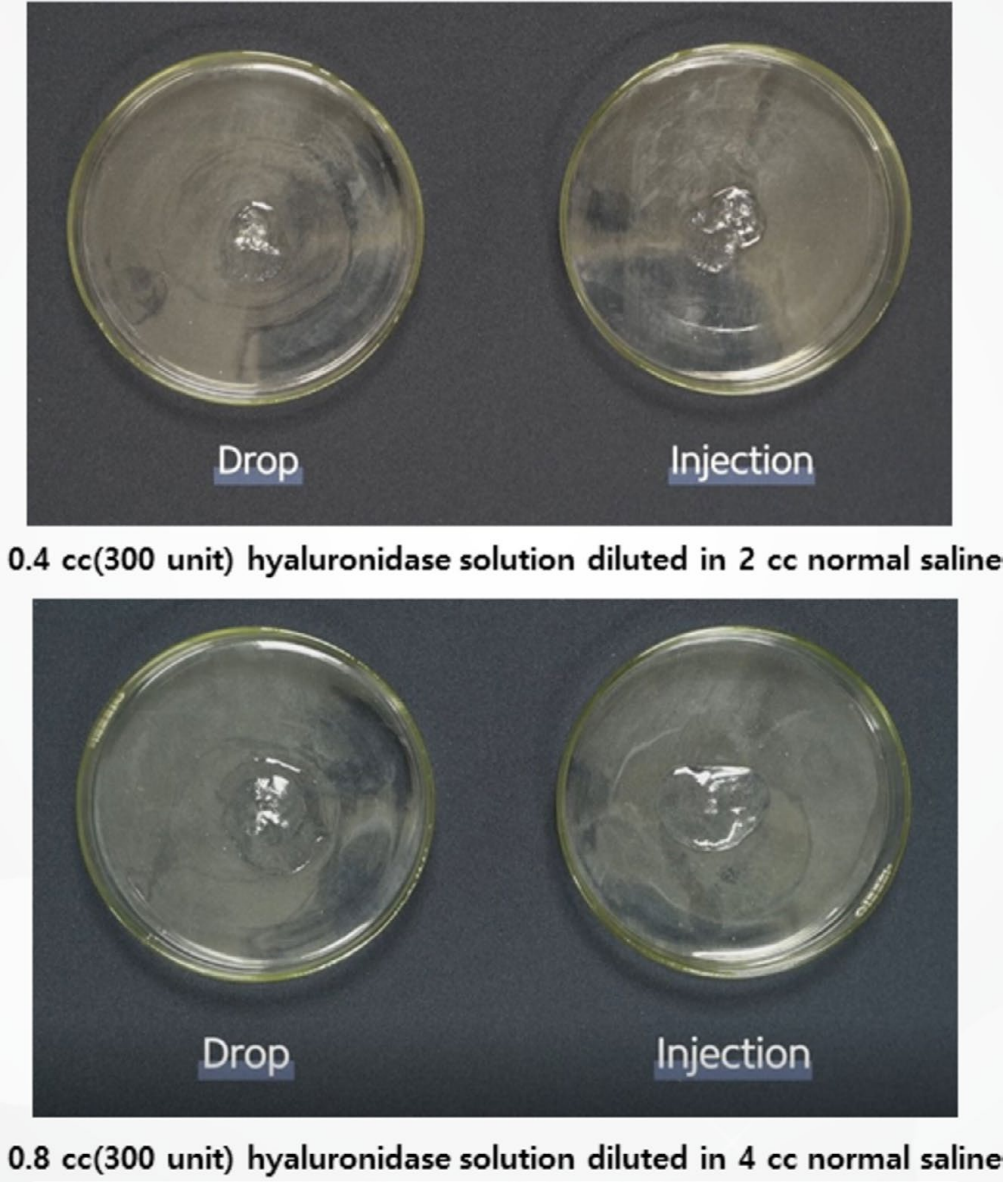
A comparison of hydration processes before and after exposure to hyaluronidase, arranged by different particle sizes of biphasic HA fillers.

The in vitro experiments evaluated the dissolution of biphasic and monophasic HA fillers using varying concentrations of hyaluronidase. Biphasic HA fillers (Restylane Lidocaine) were divided into equal masses (0.125 mL) and treated with five different hyaluronidase concentrations (112, 187.5, 300, 375, and 450 units). The hydration and dissolution rates were monitored over time (Figure 3). To assess how particle size affects dissolution, biphasic fillers with different particle sizes were also tested (Figure 4).

**FIGURE 5:**
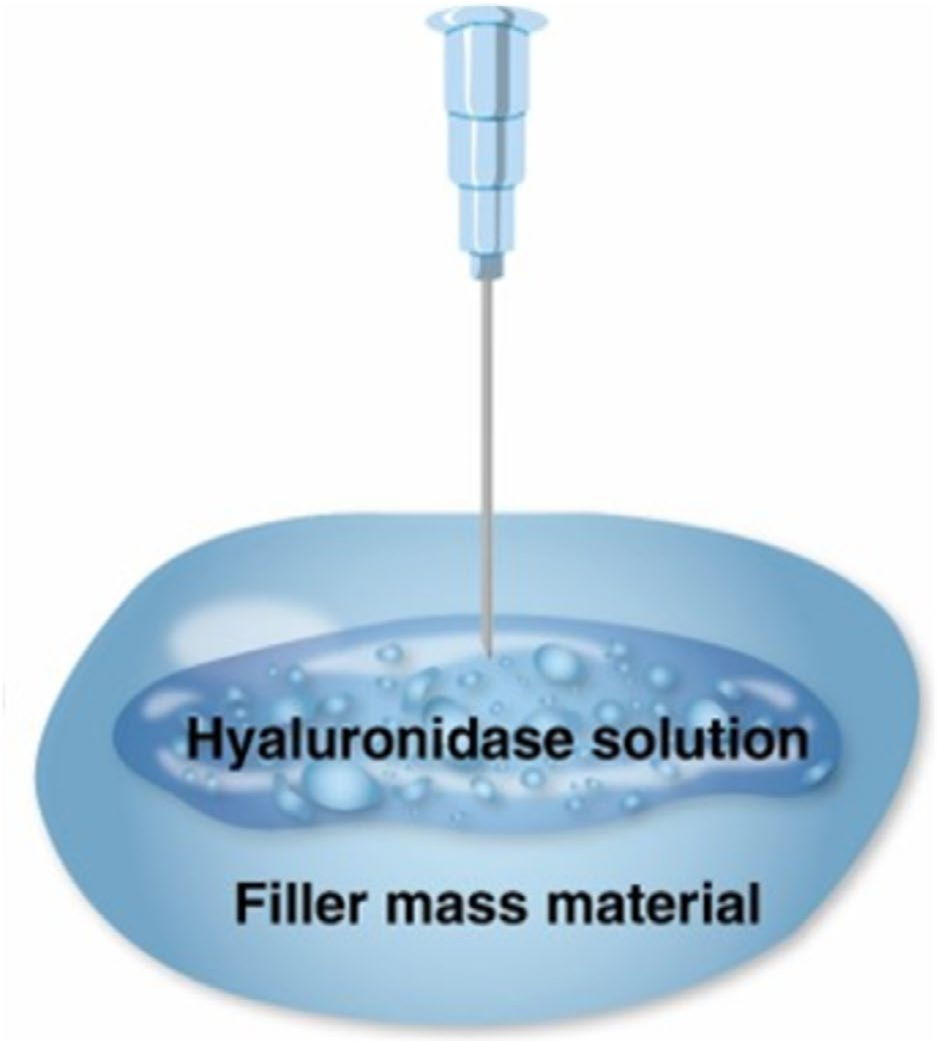
Hydration and dissolution of monophasic HA fillers treated with varying concentrations of hyaluronidase.

Monophasic fillers, which are more crosslinked, were subjected to similar treatments. They were exposed to 375 units of hyaluronidase, diluted in either 2 or 4 mL of saline, to compare the dissolution process (Figure 5). Both application techniques—surface application and direct injection—were compared for effectiveness (Figure 1).

#### 2.3.1 Results for In Vitro Dissolution of Biphasic and Monophasic Fillers

**FIGURE 6:**
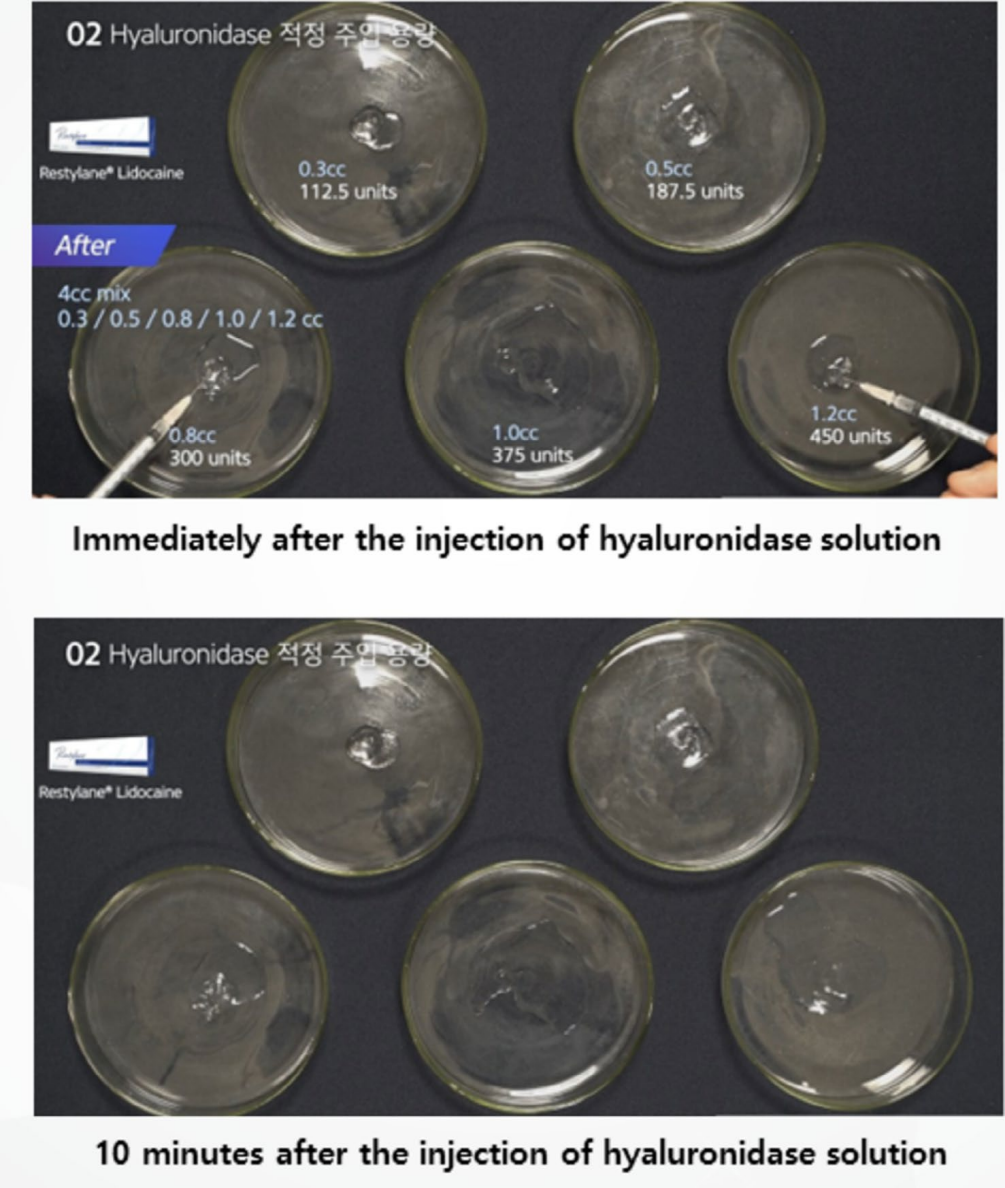
Hydration processes for biphasic HA fillers treated with varying concentrations of hyaluronidase solution.

For biphasic fillers, lower concentrations of hyaluronidase (112–187.5 units) were insufficient for full dissolution. However, higher concentrations (300–450 units) resulted in faster hydration and dissolution, especially when hyaluronidase was injected directly into the filler mass. Complete dissolution was observed within 1–2 h when 600–750 units of hyaluronidase were applied (Figure 6). Biphasic fillers with larger particle sizes required more time for dissolution (Figure 4).

**FIGURE 7:**
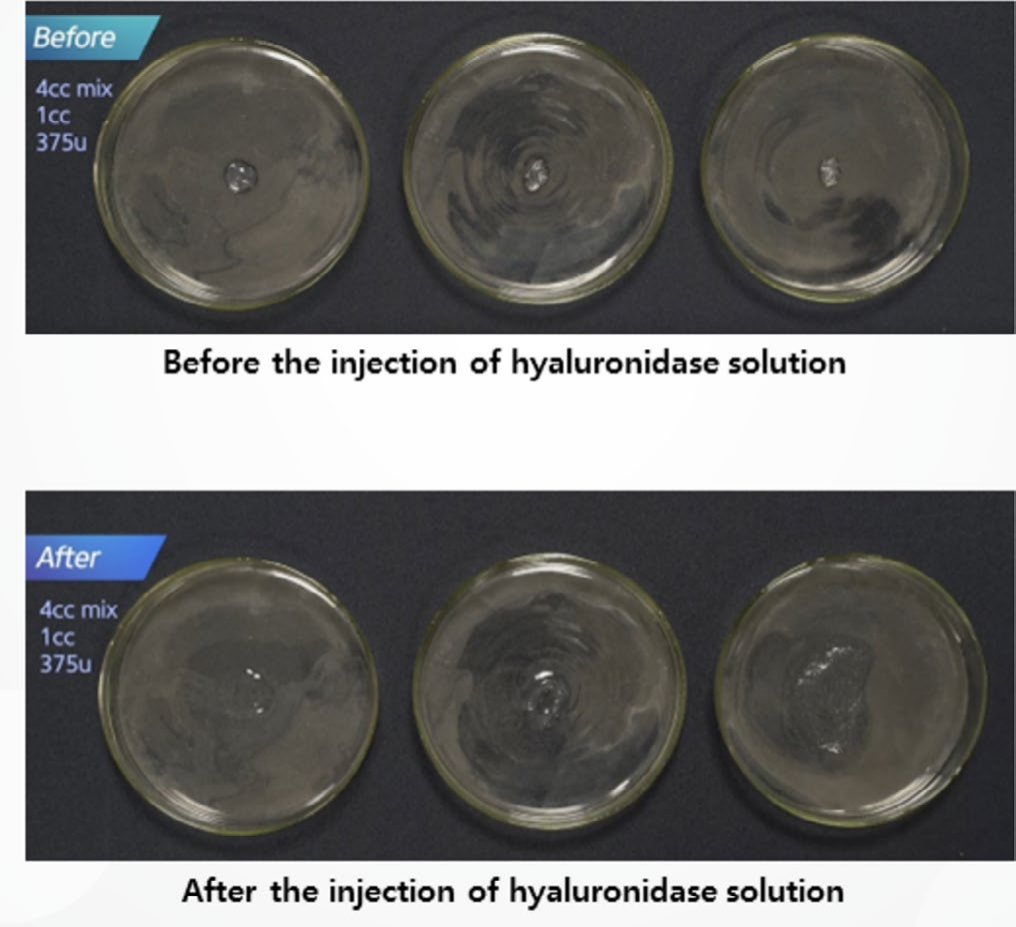
A comparison of hydration processes for different monophasic HA fillers with varying degrees of crosslinking, before and after exposure to 2 mL of diluted hyaluronidase solution.

**FIGURE 8:**
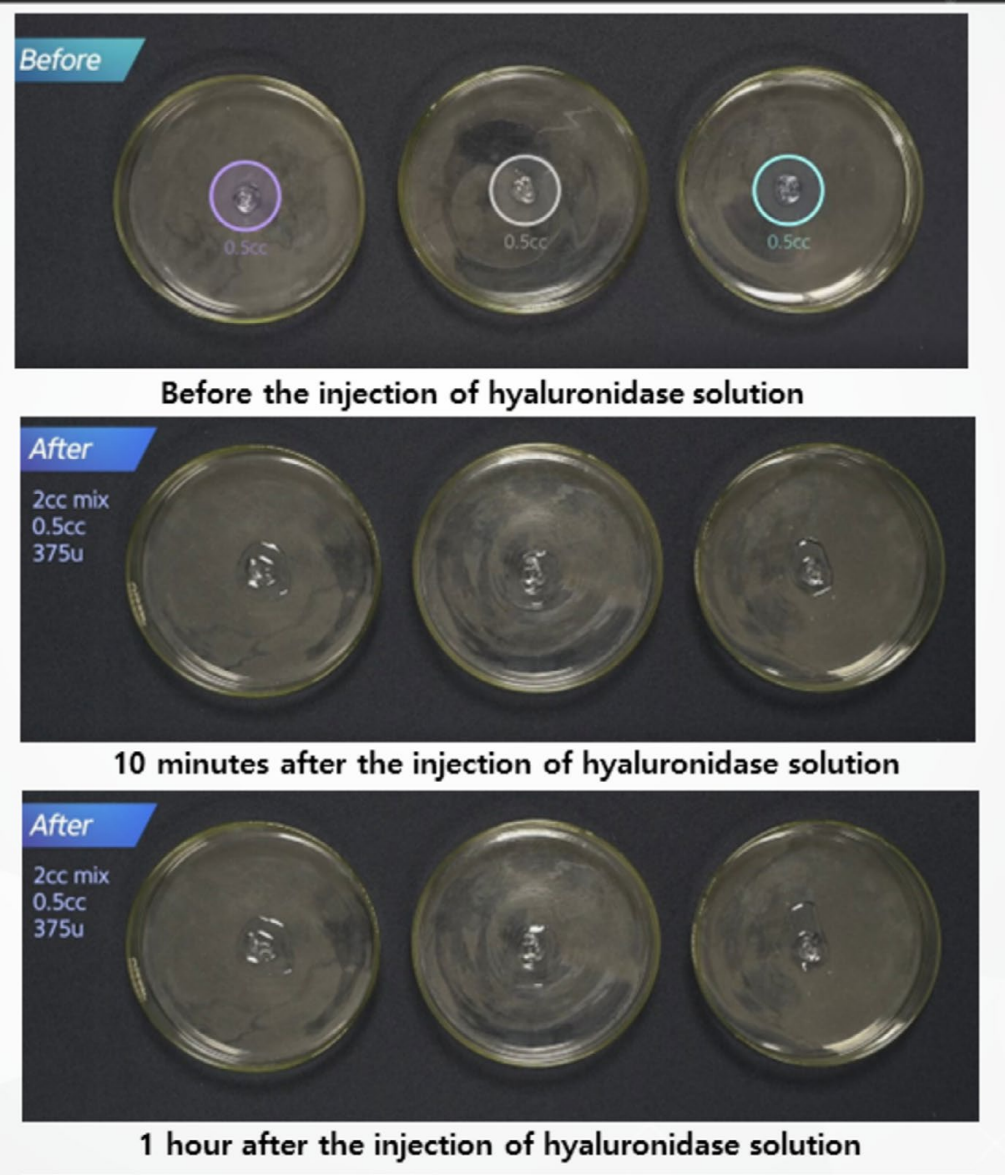
A comparison of hydration processes for different monophasic HA fillers with varying degrees of crosslinking, before and after exposure to 4 mL of diluted hyaluronidase solution.

**FIGURE 9:**
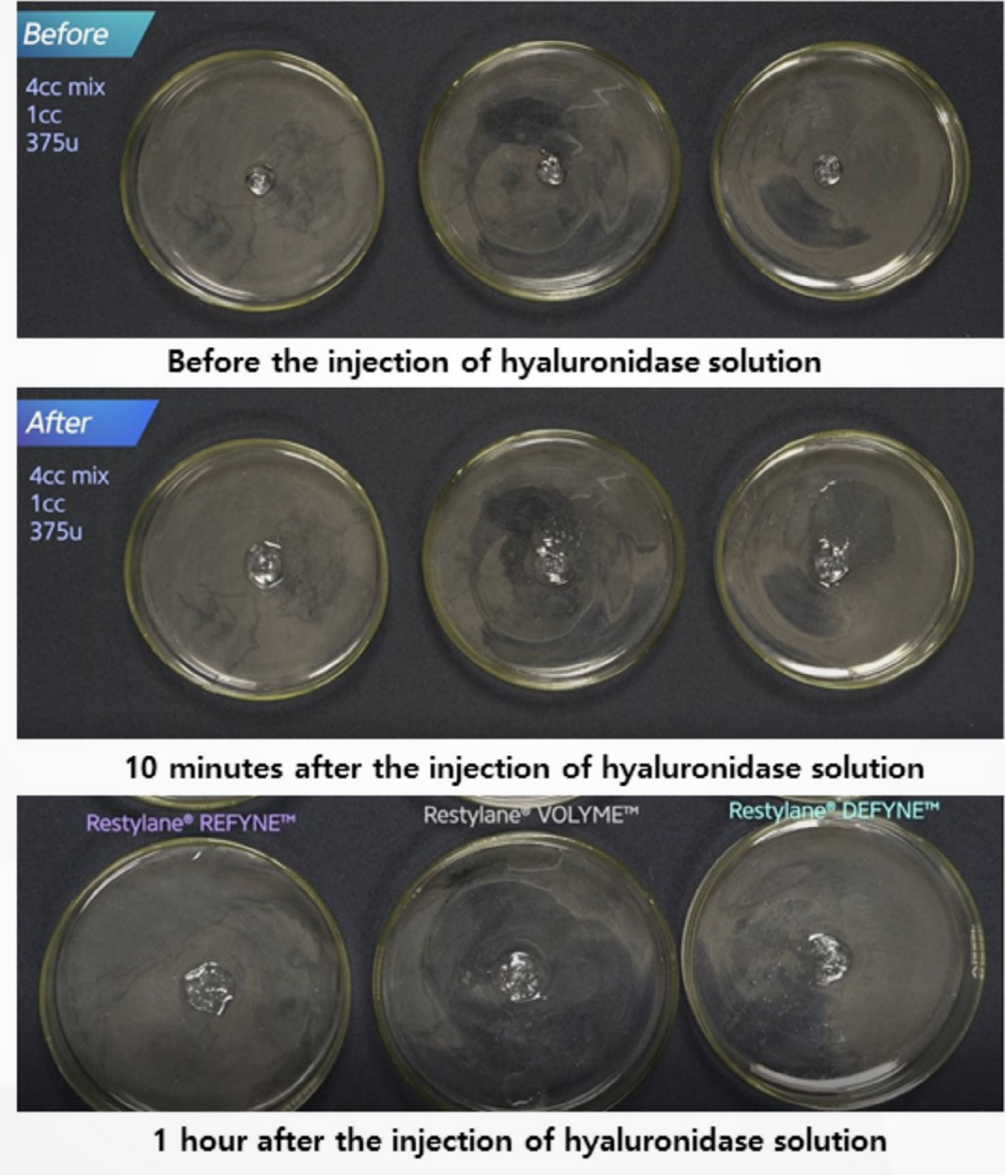
A comparison of hydration processes for different monophasic HA fillers from four companies before and after exposure to 4 mL of diluted hyaluronidase solution at 10 min and 1 h.

For monophasic fillers, which are more crosslinked, higher concentrations and longer exposure times were needed for effective dissolution. Surface application of hyaluronidase was less effective, while direct injection into the filler mass led to faster hydration and breakdown (Figures 7 and 8). Compared to biphasic fillers, monophasic fillers required higher doses of hyaluronidase and more time to break down (Figure 9).

### 2.4 Saline Control Study

**FIGURE 10:**
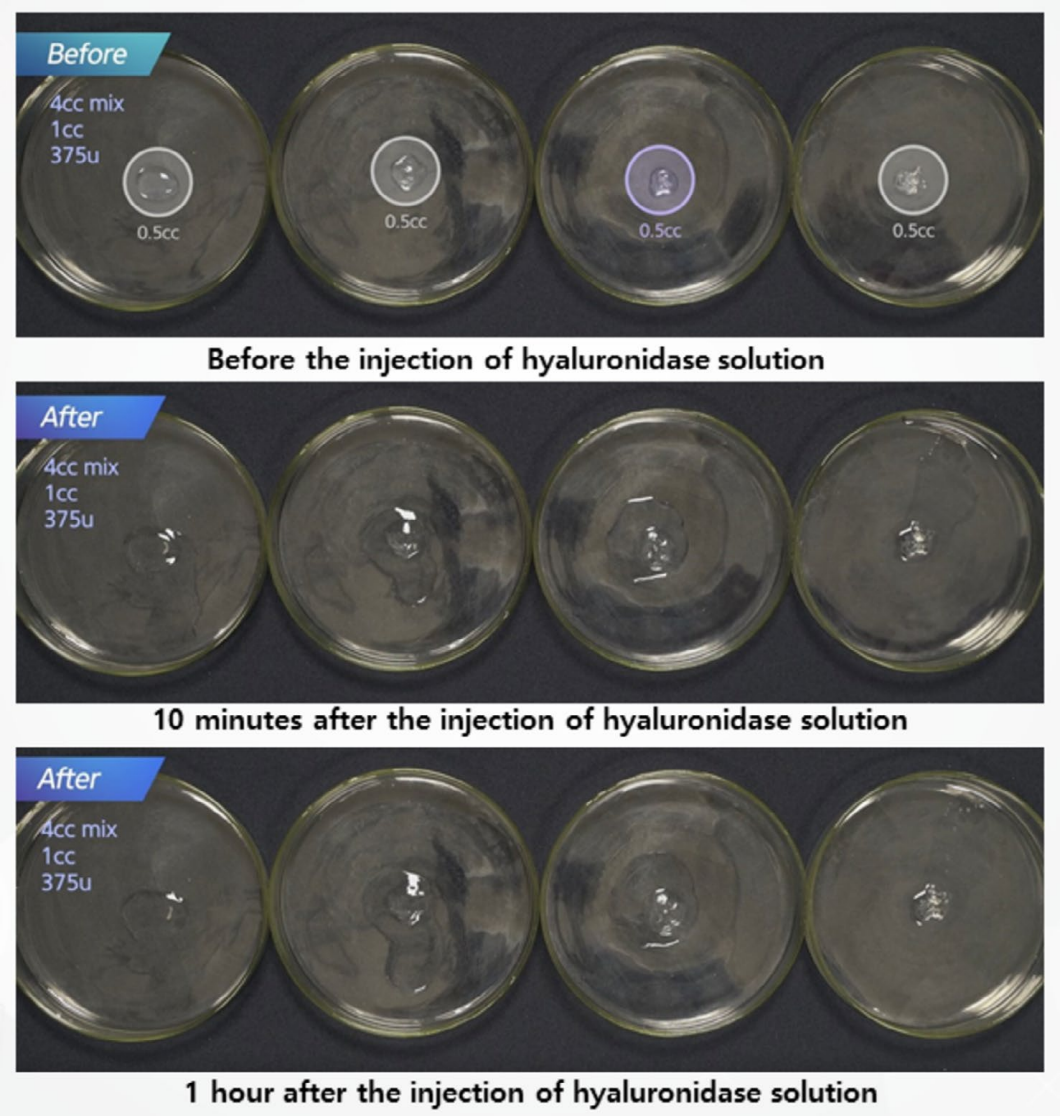
A comparison of hydration processes between monophasic and biphasic HA fillers before and after injection of normal saline.

A control study was performed to determine if saline alone could hydrate or degrade HA fillers. In this experiment, 1 mL of saline was injected into 0.5 mL of biphasic and monophasic fillers to compare the hydration process without the presence of hyaluronidase (Figure 10).

#### 2.4.1 Results for Saline Control Study

**FIGURE 11:**
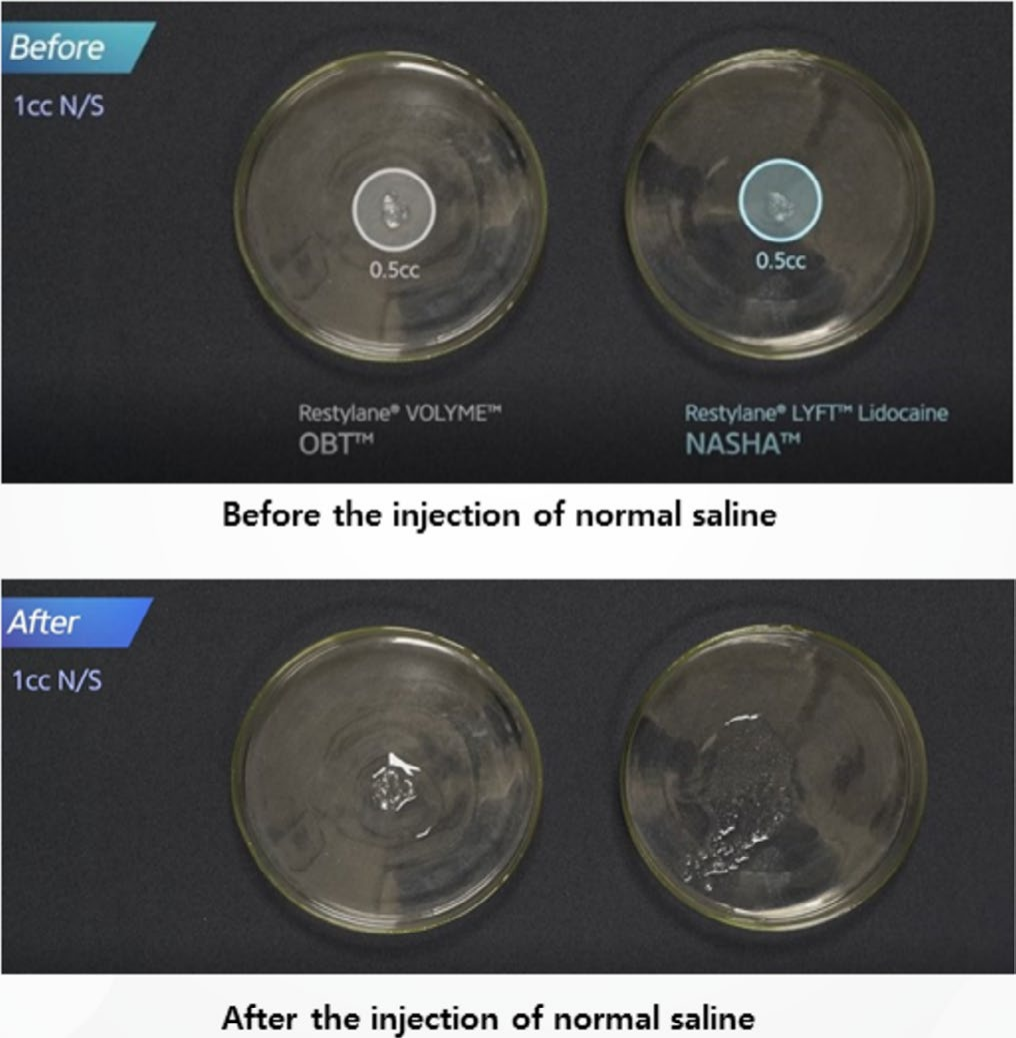
The molecular structure of hyaluronic acid and the site where hyaluronidase acts to degrade it into its constituent parts: Glucuronic acid and N‐Acetyl‐D‐glucosamine.

The control study revealed that saline alone did not lead to the dissolution of either biphasic or monophasic fillers. In biphasic fillers, saline penetrated the filler mass and caused some structural collapse, but no true degradation occurred. In monophasic fillers, the saline did not penetrate the structure effectively, leaving the filler mass mostly intact. This shows that while saline can hydrate filler particles, it is ineffective at dissolving them (Figure 11).

#### 2.4.2 Interpretation of Dissolution Experiments

The results of these experiments indicate that hyaluronidase is most effective when injected directly into the filler mass. Biphasic fillers respond more quickly to lower concentrations of hyaluronidase, while monophasic fillers, which are more crosslinked, require higher doses and longer exposure times. Clinically, the dosage and technique for hyaluronidase administration should be tailored to the specific type of filler and situation to optimize outcomes.

## 3 Discussion

The differential effects of hyaluronidase on biphasic and monophasic fillers are key considerations in clinical practice. This study demonstrates that the structural differences between these fillers significantly influence the enzyme's effectiveness. Biphasic fillers, which are less cohesive and more granular in structure, typically dissolve faster with lower concentrations of hyaluronidase. On the other hand, monophasic fillers, characterized by their smoother and more cohesive structure, require higher concentrations and longer exposure times for effective dissolution [9, 10]. This distinction is particularly crucial in emergencies such as vascular complications arising from filler injections, where rapid filler dissolution can prevent tissue necrosis [11, 12].

Understanding the properties of the specific HA filler used is essential for clinicians to optimize hyaluronidase treatment. Our results show that for biphasic fillers, approximately 500 units per 1 mL of filler is generally sufficient for effective dissolution. In contrast, monophasic fillers, due to their higher degree of crosslinking, often require up to 750 units per 1 mL, and may benefit from multiple injections and massage techniques to facilitate the dissolution process. These findings align with existing literature on the varying response rates of fillers to enzymatic breakdown [13, 14, 15, 16, 17].

One of the key findings is the importance of the injection technique. The study highlights that direct injection of hyaluronidase into the filler mass yields better results than surface application alone. This method allows the enzyme to penetrate the filler material more effectively, speeding up the hydration process and leading to more complete dissolution. Clinicians should thus prioritize direct injection techniques, particularly for monophasic fillers, where surface application alone may be insufficient.

Despite the enzyme's efficacy, it is important to consider the potential for hypersensitivity reactions. Hyaluronidase, particularly when used in high doses, can cause allergic responses. Clinicians should have antihistamines and corticosteroids readily available to manage any adverse reactions. Pretreatment may be necessary for patients with a history of allergies. Moreover, careful dosage calibration is essential to balance effective dissolution while minimizing risks [9, 14].

The intervals between hyaluronidase applications should also be tailored to individual patient needs. In vascular occlusions, repeated injections may be necessary due to the short half‐life of hyaluronidase in the bloodstream. Timely and adequate dosing is critical in these cases, with close monitoring to ensure that the filler is fully dissolved and that blood flow is restored. This study supports the recommendation of using 600–750 units of hyaluronidase in cases of vascular compromise, as well as continuous monitoring for potential adverse outcomes.

A limitation of this study is the in vitro experimental setup, which does not fully replicate the complexity of in vivo conditions. The temporary contact between hyaluronidase and the HA filler on a plate may not account for the sustained enzymatic interaction that occurs in biological tissues. Moreover, the short experimental duration of 1 h contrasts with clinical evidence indicating that hyaluronidase's effects can persist for up to 48 h postinjection. Future studies should explore longer observation periods and more complex in vivo models to better understand hyaluronidase's long‐term effects.

Clinicians must also consider the management of complications such as nodules and granulomas. The results suggest that injecting hyaluronidase directly into nodules, possibly in combination with corticosteroids and vigorous massaging, can help dissolve harder filler deposits [12]. This technique may be particularly useful for treating delayed inflammatory reactions or overcorrection of fillers.

Overall, this study underscores the importance of understanding the differences between biphasic and monophasic fillers and the necessity of tailoring the concentration of hyaluronidase, application technique, and follow‐up care to the specific clinical scenario. Our findings provide evidence that hyaluronidase is highly effective in mitigating complications from HA filler injections, provided that the appropriate technique, concentration, and precautions are followed.

## 4 Conclusion

This study demonstrates that hyaluronidase is an effective tool for managing complications from HA fillers. However, the type of filler and the application method play a crucial role in determining the enzyme's efficacy. Biphasic fillers dissolve more quickly and with lower concentrations of hyaluronidase, while monophasic fillers require higher concentrations and a more aggressive approach. Clinicians must be aware of these differences to optimize outcomes, especially in emergencies like vascular occlusion. Future studies should further investigate hyaluronidase's long‐term effects and refine clinical protocols for its use.

## Author Contributions

All authors have reviewed and approved the article for submission. Conceptualization: Gi‐Woong Hong, Kyu‐Ho Yi. Writing – Original Draft Preparation: Gi‐Woong Hong, Kyu‐Ho Yi, Kathleen Chang, Youngjin Park, Massimo Vitale, Giovanni Damiani, Kyu‐Ho Yi. Writing – Review and Editing: Jovian Wan, Gi‐Woong Hong, Kyu‐Ho Yi, Kathleen Chang, Youngjin Park, Massimo Vitale, Giovanni Damiani, Kyu‐Ho Yi. Visualization: Gi‐Woong Hong, Kyu‐Ho Yi, Hyewon Hu, Kathleen Chang, Youngjin Park, Massimo Vitale, Giovanni Damiani, Kyu‐Ho Yi. Supervision: Gi‐Woong Hong, Kyu‐Ho Yi.

## Conflicts of Interest

I acknowledge that I have considered the conflict of interest statement included in the “Author Guidelines.” I hereby certify that, to the best of my knowledge, that no aspect of my current personal or professional situation might reasonably be expected to significantly affect my views on the subject I am presenting.

## Data Availability

The data that support the findings of this study are available from the corresponding author upon reasonable request.
